# Drug repositioning: computational approaches and research examples classified according to the evidence level

**DOI:** 10.15190/d.2017.5

**Published:** 2017-06-30

**Authors:** David Vogrinc, Tanja Kunej

**Affiliations:** Department of Animal Science, Biotechnical Faculty, University of Ljubljana, Slovenia

**Keywords:** Databases, disease, drug, drug repositioning, genomics, target gene

## Abstract

Increasing need for novel drugs and their application for treating diseases are the main reasons for the development of bioinformatics platforms for drug repositioning. The use of existing approved drugs for treating other diseases reduces cost and time needed for a drug to come to clinical use. Different strategies for drug repositioning have been reported. The use of several omics types is becoming increasingly important in drug repositioning. Although there are several public databases intended for drug repositioning, not many successful cases of novel use of drugs have been reported in the literature and transferred to clinical use. Additionally, the study approaches in published literature are very heterogeneous. A classification scheme - Drug Repositioning Evidence Level (DREL) - for drug repositioning projects, according to the level of scientific evidence has been proposed previously. In the present study, we have reviewed main databases and bioinformatics approaches enabling drug repositioning studies. We also reviewed six published studies and evaluated them according to the DREL classification. The evaluated cases used drug repositioning approach for therapy of rheumatoid arthritis, cancer, coronary artery disease, diabetes, and gulf war illness. The drug repositioning study field could benefit from clearer definition in published articles therefore including drug repositioning DREL classification scheme could be included in published original and review studies. Novel bioinformatics approaches to improve prediction of drug-target interactions, continuous updating of the databases, and development of novel validation techniques are needed to facilitate the development of the drug repositioning field. Although there are still many challenges in drug repositioning and personalized medicine, stratification of patients based on their molecular signatures and testing of signature-targeting drugs should improve drug efficacy in clinical trials.

## SUMMARY

1. *Drug repositioning*
2. *Computational approaches enabling drug repositioning research*
3. *Validation strategies for drug repositioning*
4. *Examples of reports using drug repositioning approach*
5. *Conclusions and future directions*

## 1. Drug repositioning

The development of new therapeutics is essential to improve the human well-being. The standard approach to find novel drugs involves testing several thousands of compounds against a known target, to identify a lead compound^1^. These selected compounds can go through *in silico *and *in vitro *screening, before heading into the long-lasting and costly clinical trials. It takes an average of 13.5 years and 1.78 billion $, to get a drug from discovery to the market^2^. A novel approach in the development of therapeutics is to identify new applications for drugs that have already been approved. This approach is named drug repositioning and is based on the assumption, that reusing drugs that have already passed clinical trials will minimize the risk of failure in future late-stage clinical trials and thus lead to faster drug approvals^3^. There has been an increase in drug repositioning research in previous years. For example coronary artery disease^4^, diabetes^5^, gulf war illness^6^ and cancer^7^ are some of the diseases, where drug repositioning has been investigated. Examples of successful and unsuccessful cases of drug repositioning are reviewed in the paper by Li and Jones^3^. The authors of the study also classified drug repositioning into six main paths^3^. 1) Majority of repositioned drugs have been accidently identified to be effective in another disease, during the clinical testing. 2) Drugs can also have a novel activity in another disease. 3) Approved drugs can have a potential for inhibition of a certain target in another disease. 4) Repositioning can also occur when a new role is revealed for an existing target protein and a protein is found to be an important target in another disease. 5) On the metabolic level, different diseases can also share a common pathway. 6) The last path for repositioning is related to side effects that are observed in clinical trials.

Drug repositioning studies can be broadly classified into a three-step process. A primary analysis can be initiated using data from expression signatures, target biology, protein-protein or protein-small molecule network datasets and generate a list of ranked compounds for further evaluation. Secondary analysis refers to a collection of analyses approaches to filter or prioritize compounds for validation. Tertiary analyses aim to validate the compounds using experimental approaches, pre-clinical models and assess outcomes of the drug repositioning using mining of electronic health records (HER) data^8^.

**Figure 1:**
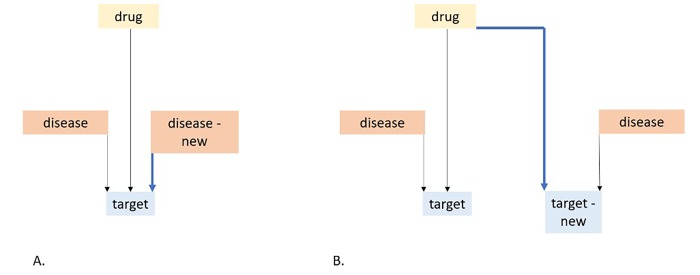
Schematic presentation of strategies for drug repositioning. A. New indication; an association between a target and a new disease. B. An association between a drug and a new target.

Genomics and similar scientific approaches, are gaining popularity in drug repositioning research. Drug - gene (target) - disease relation can serve as a framework for different drug repositioning strategies (**Figure 1**).

Bioinformatics tools and public databases, that serve for repositioning, aim to find connections between them^3^. Genomics and similar scientific approaches, are gaining popularity in drug repositioning research. Drug - gene (target) - disease relation can serve as a framework for different drug repositioning strategies (**Figure 1**). Bioinformatics tools and

Despite the fact that there are still many challenges in drug repositioning and personalized medicine research fields, stratification of patients based on their molecular signatures and testing of signature-targeting drugs is expected to become a routine approach^3^. It has also been suggested that greater improvements would come from testing different drug combinations, rather than relying only on high-throughput screening of drugs. As a disease is often seen as an integration of multiple pathologies it is therefore potentially treatable with a combination of drugs which often shows better efficacy, has fewer side effects and drug resistance is less likely^9^.

## 2. Computational approaches enabling drug repositioning research

There are many open access databases, enabling research of drug repositioning. A comprehensive literature review is an important step in drug repositioning. Majority of drug repositioning studies start with search for the literature. PubMed is one of the most popular search engines for scientific literature in the life-science. It accesses the MEDLINE database and incorporates more than 26 million citations for biomedical literature (https://www.ncbi.nlm.nih.gov/pubmed/).

Kyoto Encyclopedia of Genes and Genomes (KEGG) is an important database resource for drug repositioning. Although it is well known for its metabolic pathways presentation (KEGG PATHWAY), it also includes information about drugs (KEGG DRUG) and diseases (KEGG DISEASE) (http://www.genome.jp/kegg/). Next frequently used website is DrugBank. The DrugBank bioinformatics database combines detailed drug data (chemical structure, pharmacological and pharmaceutical function) with several drug target data (sequence, structure, and pathway) information (https://www.drugbank.ca/)^10^. It contains more than 8000 FDA approved and experimental drugs. More than 20,000 drugs and 2,360 targets are available in The Therapeutic Target Database (TTD) (http://bidd.nus.edu.sg/BIDD-Databases/ TTD/ TTD.asp)^11^. It enables the search for chemical drug information related to targets and diseases. TTD also enables search for 1,755 biomarkers, related to 365 disease conditions. The Pharmacogenomics Knowledgebase (Pharm GKB) encompasses clinical information of drugs, potential gene-drug associations and genotype-phenotype relationships (https:// www.pharmgkb.org/) and describes around 3,000 drugs and 27,000 human genes^12^. Some of the databases enable similar search opportunities. DrugBank, TTD and PharmGKB all combine disease, target gene and drug data (**Table 1**). Any of these databases can be selected for drug repositioning, using genomic approach. PubMed can be used for data mining of the literature, while KEGG is the main source for representation of metabolic pathways for drug repositioning. With the module KEGG DRUG, some information about detailed properties of the approved drugs can be found. KEGG DISEASE on the other hand, views diseases as perturbed states of the molecular system. Combination of disease and drug data, can provide visual representation of disease pathway maps.

**Table 1:** Public databases used for drug repositioning research and their properties.

Database	Artjcles	Biological pathway	Disease	Target gene	Drug data	Biomarker
PubMed	√		√		√	
KEGG		√	√		√	
DrugBank		√	√	√	√	
TTD			√	√	√	
PharmGKB	√	√	√	√	√	√

Development of novel software tools is necessary for processing of data, generated by numerous biological applications, including drug repositioning. Development of drug repositioning algorithms is important for prediction of novel drug for common diseases. Some of the recent computational advances include: pathway based repositioning, network based repositioning, protein-protein interaction driven prioritization, text-mining driven drug repositioning, protein-small molecule interactions, and protein-protein interaction driven prioritization (reviewed in Shameer et al.^8^). Additionally, improved strategies based on topological methods for network-based drug-target prediction have been developed recently^13^.

## 3. Validation strategies for drug repositioning

Computational repositioning approach enables rapid screening of candidates *in silico* and therefore reducing the number of possible repositioning candidates. However, a gold standard to perform validation of *in silico* predictions is not yet established. Brown and Patel^14^ performed a systematic review of methodologies for repositioning validation. They reviewed 39 published repositioning studies claiming validation of computational predictions. The literature analysis revealed various types of strategies for validation, which were summarized into three major types: (1) validation with a single example or case study of a single disease area, (2) sensitivity-based validation only, and (3) both sensitivity-and specificity-based validation. The authors concluded that based on currently available data, the best strategy towards the analytic validation of repositioning techniques is sensitivity-based validation.

Experts from the filed also reported that the study field could benefit from clearer definition in articles presenting drug repositioning. Therefore, a classification scheme - Drug Repositioning Evidence Level (DREL) - for different types of drug repositioning projects, according to the level of scientific evidence has been proposed^15^. DREL classification ranges from zero, which refers to predictions without experimental support, to four, which refers to drugs approved for the new indication. The authors presented a set of concepts leading to clinically efficacious repositioning hypotheses and safe applications of existing drugs. An increasing number of approaches for drug repositioning have been proposed, ranging from text mining or *in silico* screening, in vitro/ex vivo screening, study in animal disease models, and to observational studies from human trials. More precise and extensive experimental design, data preparation could lead to more effective and successful drug repositioning. DREL is a classification scheme, that can be used to evaluate drug repositioning projects according to the level of scientific evidence^15^. The authors also classified published drug repositioning studies. Example for DREL-1 classification is a manuscript reporting chemical genomic profiling for antimalarial therapies, response signatures and molecular targets^16^. Example for a DREL-2 classification is a study by Debnath *et al*.^17^, reporting phenotypic screening of approved drugs for application as amoebic dysentery therapies.

## 4. Examples of reports using drug repositioning approach

**Figure 2:**
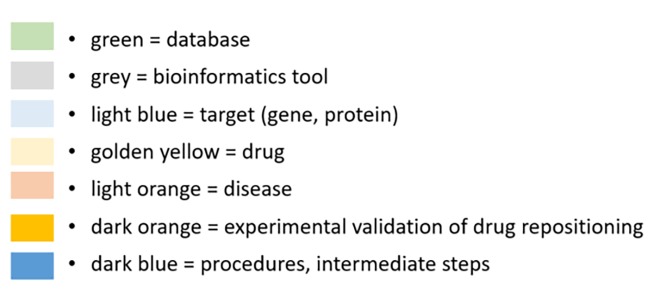
Color legend used for visualization of workflows used in evaluated drug repositioning studies. Seven data types were extracted from publications.

Published studies using drug repositioning approach are very diverse. Six examples of published projects and different search approaches for novel drugs are described in the following chapter. We evaluated the studies according to the DREL classification.**Table 2** presents classification scheme with example references scored according to the level of scientific evidence. From the literature we selected one successful repositioning study and five publications, obtained from the PubMed database using the keywords “drug repositioning”. Out of six evaluated studies, four studies were classified to the group DREL-0^4-6,18^, one to the group DREL-1^7^, and one was classified to the group DREL-4^19^. Table 2 also includes three cases^16,17,20^ classified previously by^15^. In the present study, we extracted relevant information from six published reports and sorted the data to seven data types: databases, bioinformatics tools, target (gene or protein), drug, disease, experimental approach for predicted drug reposition, and intermediate steps. Flow-charts were visualized in accordance to the presentation published previously^5^. Data types were visualized using the color legend presented in the **Figure 2**.

**Table 2:** Classification of drug repurposing studies according to the Drug Repositioning Evidence Level (DREL). Adapted from^15^.

DREL level	Quality of scientific evidence	References
0	No evidence; includes *in silico* predictions without confirmation.	^4-6,18^
1	*In vitro* studies with limited value for predicting *in vivo*/human situation.	^7,16^
2	Animal studies with hypothetical relevance in man.	^17^
3	Incomplete studies in man at the appropriate dose, e.g. proof of concept; very few cases or interference from medical records; some clinical effects observed.	^20^
4	Well-documented clinical end points observed for the repurposed drug at doses within safety limits.	^19^

### Case study 1: Rheumatoid arthritis; DREL classification = 0

**Figure 3:**
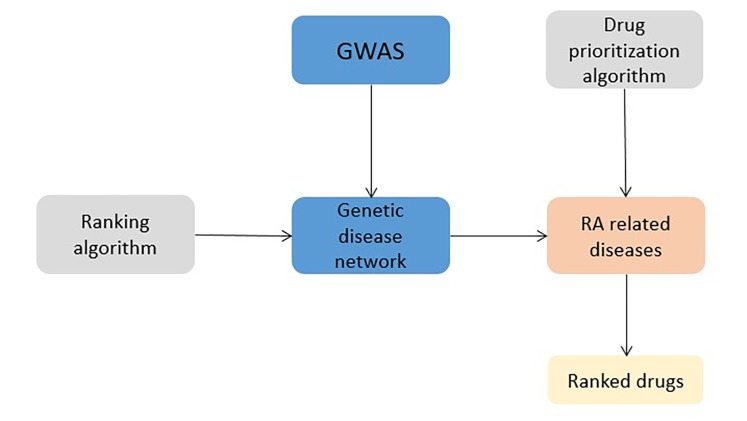
Drug repositioning study in rheumatoid arthritis 18. RA: rheumatoid arthritis, GWAS: genome-wide association study.

Xu and Wang developed two algorithms for drug repositioning in rheumatoid arthritis^19^ (**Figure 3**). A genetic disease network (GDN) was constructed using 22,470 disease-gene associations from the Catalog of Published Genome-Wide Association Studies from the US National Human Genome Research Institute (NHGRI). For evaluating of this comprehensive data, a network-based ranking algorithm was developed. A ranked list of two enriched disease classes (immune disease and autoimmune disease), consisting of 842 diseases, related with rheumatoid arthritis, was extracted from the GDN, using ranking algorithm. This data represented input for another algorithm, used for drug repositioning. Algorithm is based on a simple assumption: if a drug treats many top-ranked diseases, related with rheumatoid arthritis, it will rank higher and if a drug treats one or two lower-ranked diseases, it will rank lower. The effectiveness of the algorithm was determined, using 80 FDA approved rheumatoid arthritis drugs. Recalls, mean, and median rankings of these drugs were calculated at different ranking cutoffs (top 1%, 5%, 10%, 20%, 50%, and 100%). Drugs varied greatly from 0.04% (prednisone) to 97.83% (salicylamide), indicating that not all rheumatoid arthritis drugs can be discovered based on disease genetics. Later, the drug repositioning algorithm was used for evaluation of prediction of novel drugs for rheumatoid arthritis. A total of 165 novel drugs were obtained from the literature and clinical trials, using the drug-disease treatment knowledge bases constructed by the authors. Similar as before, precisions, recalls, and F1 measures at same ranking cutoffs as for FDA-approved drugs were calculated. The top 25 drugs had a precision of 0.89 and the best overall performance was achieved at top 10% of the drugs^18^.

### Case study 2: Diabetes; DREL classification = 0

**Figure 4:**
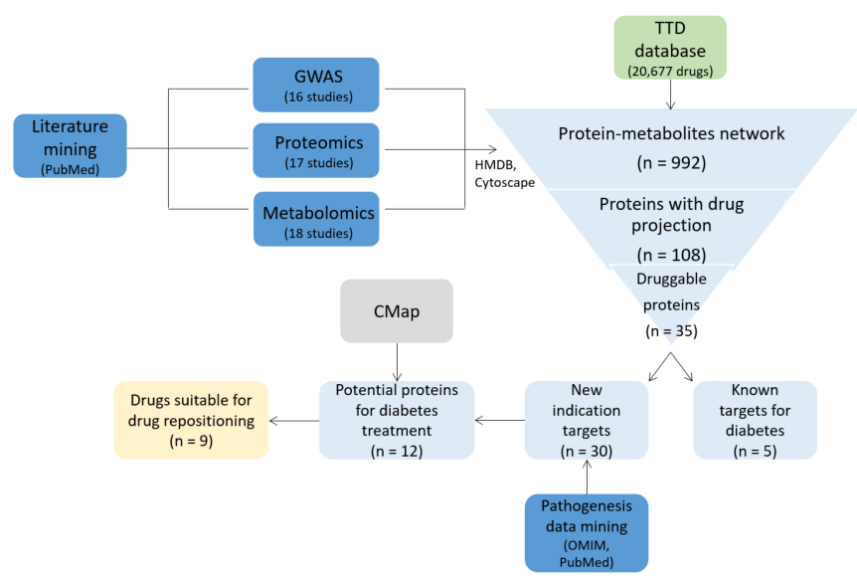
Drug repositioning study in diabetes^5^. GWAS: genome-wide association study, CMap: connectivity map, HMDB: The Human Metabolome Database, TTD: Therapeutic Target Database, OMIM: Online Mendelian Inheritance in Men. The figure is adapted after the reference^5. ^

Zhang et al. performed drug repositioning study in diabetes^5^ (**Figure 4**). They searched for the literature in PubMed, using keywords: diabetes, GWAS, proteomics, protein, metabolomics, and metabolites. 16 GWAS, 17 proteomics and 18 metabolomics papers studying diabetes were included in further research. The Human Metabolome Database (HMDB) was used to extract the names of enzymes and transporters, associated with diabetes related proteins from previous studies. Both data were combined to construct the metabolites-proteins network using Cytoscape. Furthermore, the Therapeutic Target Database (TTD) was used to evaluate whether set of diabetic risk proteins (diabetes related proteins and genes from PubMed, combined with proteins from HMDB) have a potential for drug projection. Results showed, that 108 of 992 proteins have at least one drug project. 35 out of 108 proteins were in clinical stage of approval and had no proven toxicity in human. They were used for pathogenesis data mining in Online Mendelian Inheritance in Men (OMIM) and PubMed, to gather knowledge on their loss or gain of function. Twelve protein targets, corresponding to 58 drugs, had pathogenesis information that support their potential for diabetes treatment. These drugs were assessed with Connectivity Map (CMap), a bioinformatics tool capable of finding functional connections among diseases, genetic perturbation and drug action^21^. CMap analysis indicated nine drugs suitable for drug repositioning in diabetes. Diflunisal, nabumetone, niflumic acid and valdecoxib have a common target of prostaglandin G/H synthase 2, associated with type 1 diabetes. Phenoxybenzamine and idazoxan are related with type 2 diabetes, targeting alpha-2A adrenergic receptor. The remaining three drugs are diflorasone, d-cycloserine and perhexiline.

### Case study 3: Coronary artery disease (CAD); DREL classification = 0

**Figure 5:**
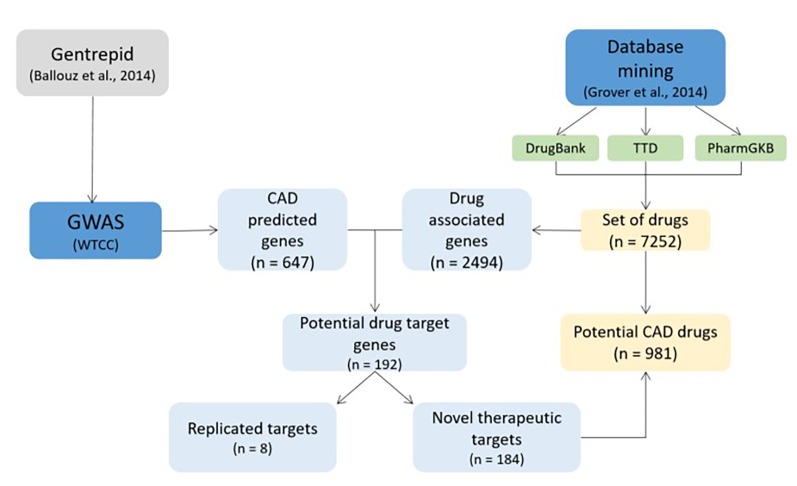
Drug repositioning study in coronary artery disease (CAD)^1,4^. GWAS: genome-wide association study, WTCC: Wellcome Trust Case Control Consortium.

Grover et al.^4^ studied GWAS data in coronary artery disease (CAD), one of the leading causes of death worldwide (**Figure 5**). Authors used Gentrepid, candidate gene prediction tool, to analyze large-scale Wellcome Trust Case Control Consortium (WTCC) GWAS study, integrating several thousand cases of CAD^22^. Gentrepid is searching for interactions and similarities between loci and is therefore particularly appropriate for analyzing the multiple loci outcomes of GWAS data^23^. 647 candidate genes for CAD were predicted using Gentrepid^4^. In their previous work Grover et al.^1^ mined three public drug databases, DrugBank, PharmGKB and TTD, to comprise a set of 7252 drugs associated with 2494 human drug targets. Predicted therapeutic targets from the predicted candidate genes were mapped with the extracted drug-gene target association files^4^. Authors were capable of distinguishing between known and novel therapeutic targets and therapeutics for CAD. 184 of 192 candidate genes associated with CAD were novel therapeutic targets; genes targeted by therapeutics already approved, or still in clinical trials for other diseases, but not for CAD. 981 drugs from all three databases were related with these genes and had potential for drug repositioning.

### Case study 4: Gulf war illness (GWI); DREL classification = 0

**Figure 6:**
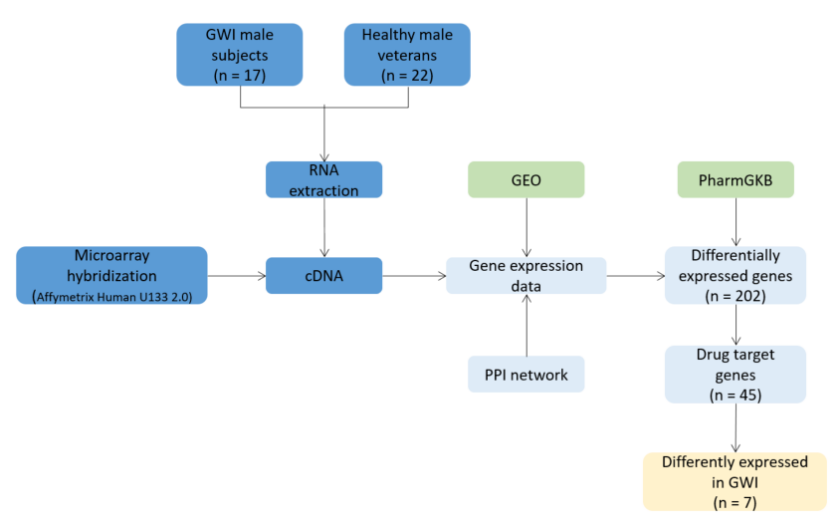
Drug repositioning study in Gulf war illness (GWI)^6^. PPI: protein-protein interaction, GEO: Gene Expression Omnibus.

Gulf war illness (GWI) is not one of the most widespread diseases worldwide, but it still gained interest in drug repositioning. A gene expression study was conducted on 17 male soldiers, suffering from GWI and 22 healthy veteran soldiers from Gulf war era^6^ (**Figure 6**). Blood samples were collected and used for RNA extraction. RNA was further converted to complementary DNA (cDNA) for hybridization, to obtain gene expression data. Data were compared to Gene Expression Omnibus (GEO) data sets and 4620 functional modules from the human protein-protein interaction (PPI) network. 202 genes from 19 modules were differentially expressed among affected and control soldiers. These genes were cross-referenced with the PharmGKB database to find gene-drug and gene-disease networks supported with pharmacogenomics research. Results showed that 45 genes were drug targets, including seven significantly different between GWI and control. The authors also compared expression modules from GWI with expression modules in other diseases. Brain, muscular and autoimmune disorders had highest similarity with GWI. Leflunomide, cisplatin, medroxprogesterone, estrogens, tamoxifen, fluvestrant and exemestane were drugs, related with rheumatoid arthritis and best potential candidates for treating GWI.

### Case study 5: Cancer; DREL classification = 1

**Figure 7:**
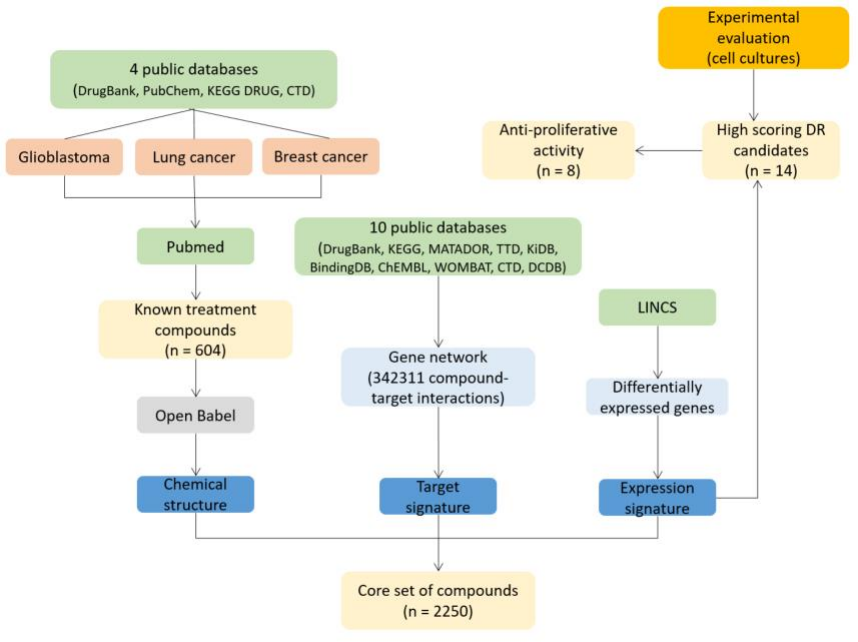
Drug repositioning study in cancer^7^. DR: drug repositioning, LINCS: Library of Integrated Network-based Cellular Signatures, CTD: Comparative Toxicogenomics Database.

Combining *in silico* predictions of possible drug repositioning candidates with experimental data for viability of various cell lines enables determination of potential novel drugs for diseases. This approach has been used to predict novel drugs for treating glioblastoma, lung cancer and breast cancer^7^ (**Figure 7**). The authors first downloaded known drug set for three types of cancer, from four public databases (DrugBank, Comparative Toxico-genomics Database, PubChem, and KEGG DRUG) and enriched data with manual search of literature on PubMed (data from 234 publications). 132, 216 and 256 compounds for the treatment of glioblastoma, lung cancer, and breast cancer were found, respectively. Researchers additionally collected 1155 compounds, showing anti-proliferative activity against any other cancer type (including drugs from known drug, associated with other types of cancer apart from glioblastoma, lung cancer, and breast cancer). Their chemical structure was determined using Open Babel. Ten public databases were used to construct network of genes, associated witch each drug for target signature. Expression signature consisted of different gene-expression profiles, generated by the Library of Integrated Network-based Cellular Signatures (LINCS). LINCS produced network of differentially expressed genes of cancer cell lines, due to the treatment with more than 20,000 chemical compounds^24^. Structure, target and expression signature served for the construction of a series of classifiers for drug reposition prediction. 14 high-scoring candidates solely based on expression signatures were predicted. They were chosen for experimental evaluation of their anti-tumor activity, using four glioblastoma cell lines and eight patients-derived primary cells. Eight out of 14 compounds inhibited growth of the cells significantly at the concentration of 10 µM: ivermectin, trifluridine, astemizole, amlodipine, maprotiline, apomorphine, mometasone, and notriptyline.

### Case study 6: Anti-HIV drugs for cancer therapeutics; DREL classification = 4

**Figure 8:**
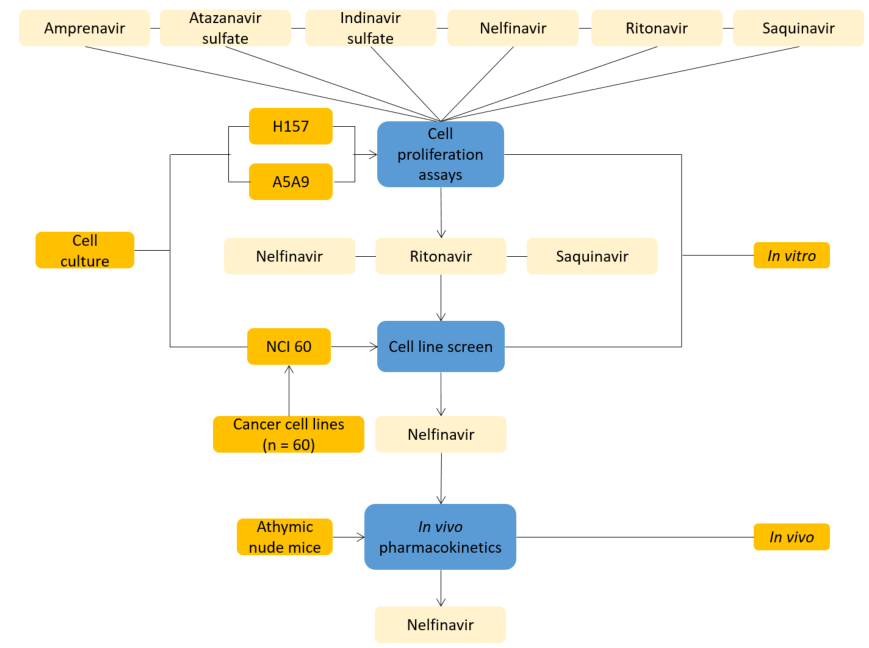
Drug repositioning study of anti-HIV drugs for cancer therapeutics^19^. H157, A5A9: human non – small cell lung cancer cell lines.

Six anti-HIV drugs were tested against a panel of 60 cancer cell lines using cellular proliferation assays and nelfinavir was found to be a potent broad-spectrum anti-tumor agent^19^ (Figure 8). Nelfinavir has entered at least eight cancer clinical trials^3^.

## 5. Conclusions and future directions

Drug repositioning is a strategy, which facilitates transfer of new candidate therapies from research into health care. The availability of high-performance computing and various databases have enhanced the ability to set testable hypotheses for drug repositioning. However, continuous updating of databases, development of novel bioinformatics softwares and validation approaches could further accelerate the progress of the study field. Additionally, classification of drug repositioning projects according to the validation level would enable better overview of the current knowledge and more targeted planning of future experiments. DREL scheme enables evaluation of drug repositioning projects according to the level of scientific evidence from 0 to 4. However, studies scored with DREL-0 should not be underestimated, because they often present novel bioinformatics approaches for drug repositioning and new predicted connections for further experimental validation. Individualized network-based drug repositioning showed promise in the development of precision medicine. Although there are still many challenges in drug repositioning and personalized medicine, stratification of patients based on their molecular disease signatures and testing of signature-targeting drugs should improve drug efficacy in clinical trials. Characterization of a patient’s genetic profile is expected to become a routine approach for diagnosing diseases and for recommending the most appropriately targeted therapy to an individual patient.

- **Drug repositioning enables expansion of the therapeutic application of drugs**
- **Including a Drug Repositioning Evidence Level (DREL) classification scheme in original and review studies would enable better overview of the current knowledge, more efficient planning of future experiments and therefore facilitate research developments in the drug repositioning study field.**
- **Novel bioinformatics approaches to improve prediction of drug-target interactions, continuous updating of the databases and development of novel validation techniques are needed to facilitate the development of the drug repositioning field.**
